# Comparative associations of the TyG index and METS-IR with arterial stiffness in women: a cross-sectional analysis by menopausal status

**DOI:** 10.3389/fendo.2026.1902406

**Published:** 2026-08-19

**Authors:** Like Yang, Yutao Wang, Ruofei Han, Kundi Lv, Xue Pang, Kuiquan Song, Yanxia Hu

**Affiliations:** 1 First Clinical Medical College, Shandong University of Traditional Chinese Medicine, Jinan, China; 2 Department of Peripheral Vascular Surgery, Guang’anmen Hospital Jinan Hospital, China Academy of Chinese Medical Sciences (Jinan Municipal Hospital of Traditional Chinese Medicine), Jinan, China; 3 Department of Colorectal Surgery, The First Affiliated Hospital of Shandong First Medical University, Jinan, China; 4 Department of General Affairs, Jinan Center for Disease Control and Prevention, Jinan, China

**Keywords:** arterial stiffness, brachial-ankle pulse wave velocity, menopause, metabolic score for insulin resistance, triglyceride-glucose index

## Abstract

**Objective:**

To compare the cross-sectional associations of the triglyceride–glucose (TyG) index and the metabolic score for insulin resistance (METS-IR) with brachial–ankle pulse wave velocity (baPWV) in women and to examine whether these associations differed by menopausal status.

**Methods:**

This cross-sectional secondary analysis included 306 women (134 premenopausal and 172 postmenopausal). baPWV was analyzed as a continuous measure of arterial stiffness. Associations with TyG and METS-IR were examined using Pearson correlation, restricted cubic splines, and multivariable linear regression; the primary adjusted model included age, menopausal status, systolic blood pressure, uric acid, LDL-C, and eGFR and omitted BMI because BMI is a component of METS-IR. Exploratory ROC analyses compared the discrimination of covariate-adjusted TyG and METS-IR models for elevated baPWV (≥1400 cm/s) using the DeLong test. Sensitivity and menopausal-status subgroup analyses were also performed.

**Results:**

Both METS-IR and TyG were positively correlated with baPWV in unadjusted analyses. After adjustment for menopausal status, age, SBP, uric acid, LDL-C, and eGFR, METS-IR remained associated with baPWV (β = 4.72, 95% CI 1.03–8.41, P = 0.012), whereas TyG did not (β = 26.66, 95% CI −11.49–64.81, P = 0.170). In stratified analyses, METS-IR was associated with baPWV in postmenopausal women (β = 5.68, 95% CI 0.74–10.61, P = 0.025) but not in premenopausal women (β = 4.16, 95% CI −1.22–9.53, P = 0.128); however, the interaction was not significant (P for interaction = 0.722). The TyG and METS-IR models showed similar exploratory discrimination for elevated arterial stiffness (AUC 0.831 *vs* 0.834; DeLong P = 0.331).

**Conclusion:**

In this cross-sectional dataset of women, METS-IR retained an adjusted association with baPWV, whereas the corresponding association for TyG was not statistically significant. Menopausal status did not significantly modify either association. The TyG and METS-IR models showed similar exploratory discrimination for elevated baPWV. Prospective external studies are needed to assess temporal associations and clinical utility.

## Introduction

Atherosclerosis is a progressive disease characterized by the accumulation of lipids, inflammatory cells, and fibrous elements in the arterial walls. It is a leading cause of coronary artery disease, stroke, and peripheral vascular disease, which collectively contribute to high global morbidity and mortality rates (1). The pathogenesis involves endothelial dysfunction, lipid infiltration, and smooth muscle cell proliferation (2). Identifying early-stage atherosclerosis before the onset of severe complications is crucial, as it is often asymptomatic (3). Arterial stiffness is an early indicator of vascular aging and a robust predictor of adverse clinical events and mortality (4). The brachial-ankle pulse wave velocity (baPWV) is a validated, non-invasive indicator of arterial stiffness. Elevated baPWV indicates an increased atherosclerotic burden, making the identification of its determinants critical for early risk stratification (5).

Cardiovascular risk profiles change dynamically throughout life, particularly among women. The cardiovascular risk in women increases significantly during and after menopause (6). The abrupt decline in estrogen levels during the menopausal transition causes endothelial dysfunction and metabolic deterioration (7). These physiological changes result in a rapid increase in arterial stiffness, which often equals or surpasses that of age-matched men in later life (8). During this critical period, traditional risk assessment tools primarily focus on conventional risk factors and often fail to detect subclinical vascular changes in women (9). Therefore, novel and sensitive biomarkers capable of detecting early metabolic and vascular shifts are required in the female population.

Insulin resistance (IR) is a primary driver of metabolic dysfunction and arterial stiffening. Although the hyperinsulinemic-euglycemic clamp is the gold standard for evaluating IR, it is highly invasive and expensive. The triglyceride-glucose (TyG) index has recently been proposed as a reliable, non-insulin-based surrogate marker for IR (10). Similarly, the metabolic score for IR (METS-IR) has emerged as another easily calculated index (11). Previous epidemiological studies have indicated that TyG and METS-IR are associated with higher baPWV and an elevated risk of atherosclerotic disease in the general population (10–12).

Although both indices have been associated with arterial stiffness, their comparative cross-sectional associations with baPWV in women and potential effect modification by menopausal status remain unclear. We therefore compared the associations of TyG and METS-IR with baPWV and explored whether these associations differed by menopausal status. We also performed an exploratory comparison of the discrimination of covariate-adjusted models containing either index for elevated baPWV.

## Materials and methods

### Study population

The present study was a secondary analysis of de-identified data from a previously published cross-sectional study conducted at Murakami Memorial Hospital (13). According to the source publication, the original study was approved by the Ethics Committee of Murakami Memorial Hospital and was conducted in accordance with the Declaration of Helsinki. All participants provided written informed consent. The ethics approval number was not reported in the source publication or the publicly available dataset. Because the present analysis used de-identified, publicly available data and involved no new participant recruitment or data collection, no additional participant consent was required for this secondary analysis. Initially, data for 307 female participants were extracted. After excluding one participant with significant missing data, a final cohort of 306 women was included in the analysis. The participants were stratified by menopausal status into pre-menopausal (n = 134) and post-menopausal (n = 172) groups.

### Data collection and measurements

Demographic and clinical data, including age, body mass index (BMI), systolic blood pressure (SBP), and diastolic blood pressure (DBP), were obtained from the repository. baPWV and ankle-brachial index (ABI) were measured using an automatic waveform analyzer (Colin Medical Technology, Komaki, Japan). Venous blood samples were collected after an 8-h fast to determine biochemical parameters, including aspartate aminotransferase (AST), alanine aminotransferase (ALT), fasting blood sugar (FBS), uric acid (UA), total cholesterol (TC), triglycerides (TG), high-density lipoprotein (HDL), and low-density lipoprotein (LDL). The estimated glomerular filtration rate (eGFR) was calculated using the three-variable Japanese equation, with a correction factor of 0.739 applied to female participants (14). The atherogenic index of plasma (AIP) was calculated as log10[TG (mmol/L)/HDL-C (mmol/L)] (15). The TyG index was calculated as ln[TG (mg/dL) × FBS (mg/dL)/2] (16), and METS-IR was calculated as ln[2 × FBS (mg/dL) + TG (mg/dL)] × BMI (kg/m²)/ln[HDL-C (mg/dL)] (17).

### Statistical analysis

Continuous variables were assessed for normality using the Shapiro–Wilk test. Normally distributed variables are presented as mean ± standard deviation and were compared using the independent-samples Student’s t-test; non-normally distributed variables are presented as median (interquartile range) and were compared using the Mann–Whitney U test. Pearson correlation coefficients were calculated to describe the unadjusted associations of TyG and METS-IR with baPWV, and scatter plots with fitted regression lines and 95% confidence intervals were used for visualization.

Restricted cubic spline regression with three knots at the 10th, 50th, and 90th percentiles was used to examine potential nonlinear associations, with the median of each index as the reference. Multivariable linear regression was used to estimate β coefficients and 95% confidence intervals. Model 1 was unadjusted, Model 2 was adjusted for age, and Model 3 was adjusted for age, menopausal status, SBP, uric acid, LDL-C, and eGFR. BMI was excluded from the primary adjusted models because it is a component of METS-IR. Variance inflation factors were calculated to assess multicollinearity.

For exploratory discrimination analyses, elevated arterial stiffness was defined as baPWV ≥1400 cm/s according to previously established criteria (18, 19). Two multivariable logistic regression models included age, SBP, uric acid, LDL-C, eGFR, and menopausal status, with either TyG or METS-IR added separately. AUCs and 95% confidence intervals were calculated and compared using the DeLong test. Sensitivity analyses excluded participants with either fasting blood sugar >126 mg/dL or SBP >140 mmHg.

No *a priori* sample-size calculation was performed because this was a secondary analysis of an existing public dataset. All eligible women with complete data for the prespecified variables were included. Estimates are presented with 95% confidence intervals, and the spline, subgroup, sensitivity, and ROC analyses were considered exploratory. All tests were two-sided, with P<0.05 considered statistically significant.

All analyses were performed in Python version 3.12.13 using NumPy 2.3.5, pandas 2.2.3, SciPy 1.17.0, and scikit-learn 1.8.0. Restricted cubic spline analyses and the DeLong test were implemented using prespecified Python routines, and figures were generated using Matplotlib 3.10.8.

## Results

### Baseline characteristics of the participants

**Table 1** summarizes the baseline characteristics of the 306 female participants, stratified by menopausal status. Compared with pre-menopausal women, post-menopausal women were older and had higher SBP, FBS, UA, TC, TG, LDL-C, TyG, METS-IR, AIP, and baPWV, together with lower eGFR (all P<0.05). BMI, DBP, HDL-C, and ABI did not differ significantly between the groups (**Table 1**).

**Table 1:** Baseline characteristics of the study population stratified by menopausal status.

Variables	Pre-menopause (n = 134)	Post-menopause (n = 172)	Statistic	*P*
Age (years)	44.67 ± 6.17	58.06 ± 6.05	t = –19.05	<0.001
BMI (kg/m^2^)	22.18 ± 3.10	21.92 ± 2.69	t = 0.78	0.437
SBP (mmHg)	113.26 ± 14.93	117.02 ± 14.65	t = –2.21	0.028
DBP (mmHg)	70.50 ± 9.57	72.28 ± 9.30	t = –1.64	0.102
METS-IR	29.21 ± 4.53	30.71 ± 6.18	t = –2.46	0.015
TyG	7.80 ± 0.64	8.04 ± 0.59	t = –3.42	<0.001
AST (U/L)	17.00 (15.00, 20.00)	19.00 (16.00, 23.00)	Z = –3.50	<0.001
ALT (U/L)	15.00 (11.00, 19.00)	16.00 (13.00, 19.00)	Z = –2.30	0.021
FBS (mg/dL)	90.50 (86.00, 96.00)	95.00 (89.00, 101.00)	Z = –4.21	<0.001
UA (mg/dL)	3.90 (3.30, 4.60)	4.10 (3.60, 4.70)	Z = –2.08	0.037
TC (mg/dL)	192.50 (172.25, 217.75)	227.50 (206.00, 247.25)	Z = –7.73	< 0.001
TG (mg/dL)	52.00 (36.00, 75.25)	62.50 (48.00, 93.00)	Z = –3.28	0.001
HDL (mg/dL)	59.00 (50.18, 69.30)	61.90 (53.38, 69.82)	Z = –1.53	0.125
LDL-C (mg/dL)	116.00 (95.25, 131.75)	138.85 (114.30, 161.00)	Z = –6.51	< 0.001
eGFR (mL/min/1.73m^2^)	76.34 (68.75, 84.08)	67.05 (64.00, 77.05)	Z = –5.58	< 0.001
ABI	1.14 (1.12, 1.20)	1.16 (1.12, 1.20)	Z = –1.66	0.096
baPWV (cm/s)	1252.50 (1133.25, 1352.75)	1438.00 (1303.00, 1581.50)	Z = –7.87	< 0.001
AIP	-0.45 (-0.63, -0.25)	-0.35 (-0.52, -0.15)	Z = –2.25	0.024

### Correlations between novel IR indices and arterial stiffness

**Figure 1:**
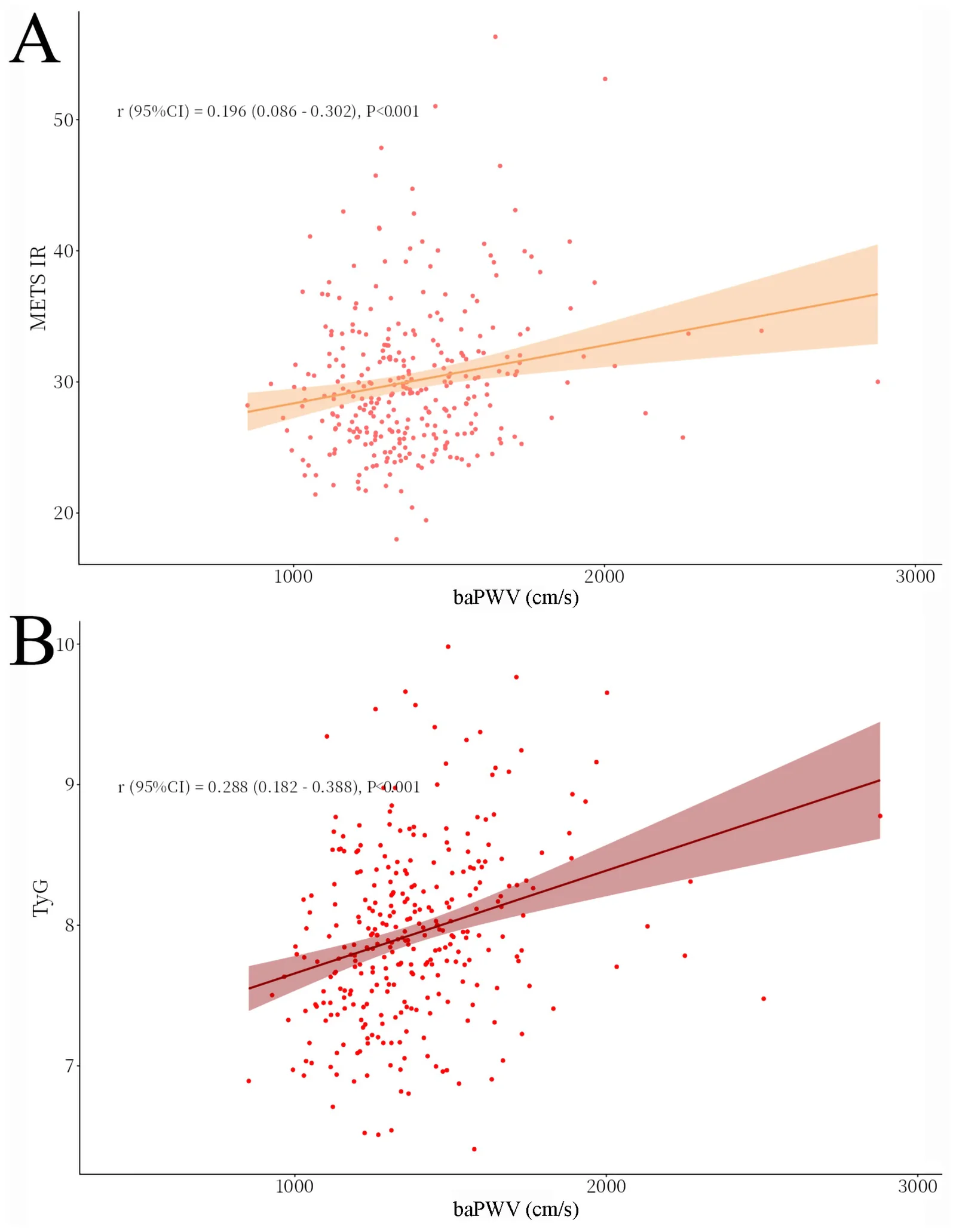
Scatter plots showing the unadjusted correlations between baPWV and **(A)** METS-IR and **(B)** the TyG index. Solid lines represent fitted linear regression lines, and shaded areas represent 95% confidence intervals. METS-IR, Metabolic score for insulin resistance; TyG, Triglyceride-glucose; baPWV, Brachial-ankle pulse wave velocity; CI, Confidence interval.

Both TyG (*r* = 0.288, *P* < 0.001) and METS-IR (*r* = 0.196, *P* < 0.001) were positively correlated with baPWV in unadjusted analyses (**Figure 1**).

### Dose-response relationships between novel IR indices and baPWV

**Figure 2:**
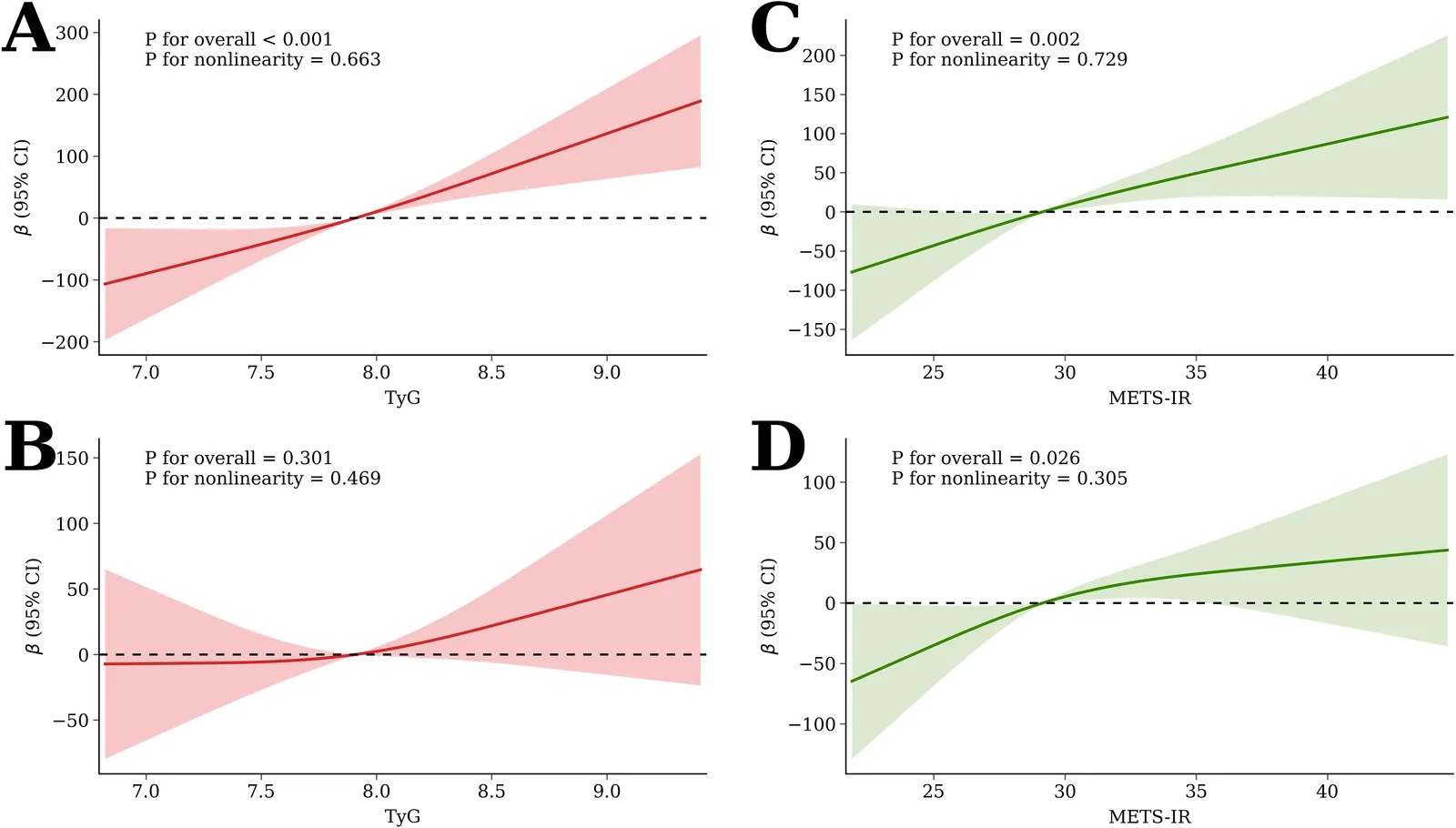
Restricted cubic spline curves illustrating the associations of the TyG index and METS-IR with baPWV. **(A)** Unadjusted and **(B)** fully adjusted models for the TyG index. **(C)** Unadjusted and **(D)** fully adjusted models for METS-IR. The fully adjusted models included age, menopausal status, systolic blood pressure, uric acid, LDL-C, and eGFR. The solid lines represent estimated β coefficients, and the shaded areas represent 95% confidence intervals. The horizontal dashed line indicates the reference value (β = 0). Three knots were placed at the 10th, 50th, and 90th percentiles, with the median used as the reference.

After full adjustment, the overall association between the TyG index and baPWV was not statistically significant (P for overall = 0.301; P for nonlinearity = 0.469). In contrast, METS-IR remained positively associated with baPWV (P for overall = 0.026), with no evidence of nonlinearity (P for nonlinearity = 0.305; **Figure 2**).

### Independent associations of novel IR indices with baPWV

Both indices were positively associated with baPWV in the unadjusted model (TyG: *β* = 113.73, 95% CI 71.12–156.34, P < 0.001; METS-IR: *β* = 8.67, 95% CI 3.78–13.57, *P* < 0.001) and after adjustment for age (TyG: *β* = 73.70, 95% CI 37.09–110.31, *P* < 0.001; METS-IR: *β* = 6.79, 95% CI 2.71–10.86, *P* = 0.001). In Model 3, which adjusted for age, menopausal status, systolic blood pressure, uric acid, LDL-C, and eGFR, METS-IR remained positively associated with baPWV (*β* = 4.72, 95% CI 1.03–8.41, *P* = 0.012), whereas the association for TyG was not statistically significant (*β* = 26.66, 95% CI −11.49–64.81, *P* = 0.170; **Table 2**). All variance inflation factors were <3.0.

**Table 2:** Multivariable linear regression analysis evaluating the associations between novel IR indices and baPWV.

Variables	Model 1		Model 2		Model 3	
	β (95% CI)	*P*	β (95% CI)	*P*	β (95% CI)	*P*
TyG	113.73 (71.12,156.34)	< 0.001	73.70 (37.09,110.31)	< 0.001	26.66 (–11.49,64.81)	0.170
METS-IR	8.67 (3.78,13.57)	< 0.001	6.79 (2.71,10.86)	0.001	4.72 (1.03,8.41)	0.012

### Exploratory comparison of discrimination

**Figure 3:**
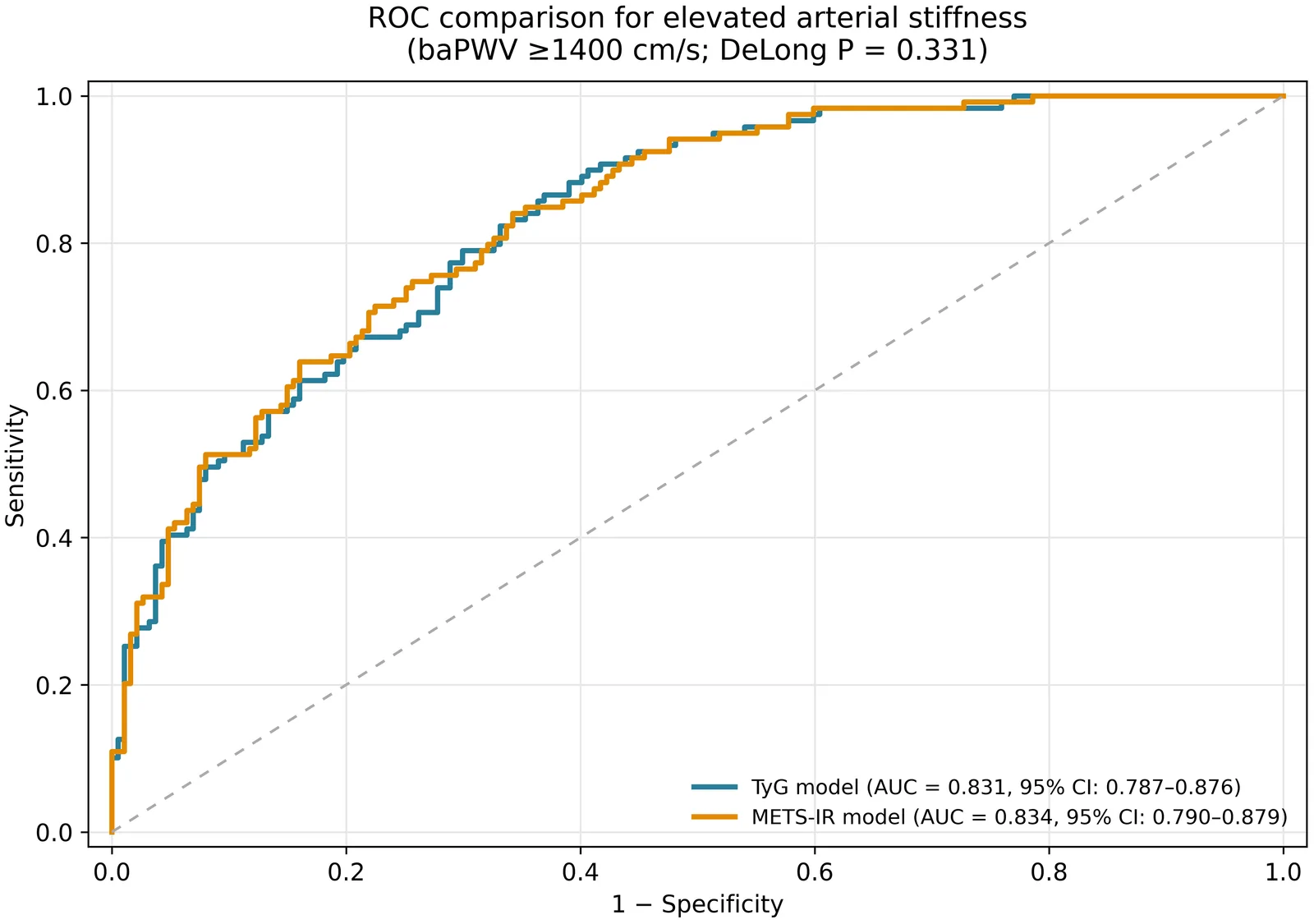
Receiver operating characteristic curves comparing the exploratory discrimination of the TyG and METS-IR models for elevated arterial stiffness. Elevated arterial stiffness was defined as baPWV ≥1400 cm/s. Both models included age, systolic blood pressure, uric acid, LDL-C, eGFR, and menopausal status, with either TyG or METS-IR added separately. AUC, area under the curve; CI, confidence interval; baPWV, brachial–ankle pulse wave velocity.

The covariate-adjusted models containing TyG or METS-IR showed similar discrimination for elevated arterial stiffness. The AUC was 0.831 (95% CI 0.787–0.876) for the TyG model and 0.834 (95% CI 0.790–0.879) for the METS-IR model; the difference was not statistically significant (DeLong P = 0.331; **Figure 3**).

### Sensitivity analysis

After excluding participants with either fasting blood sugar >126 mg/dL or systolic blood pressure >140 mmHg, the directions of association remained positive, but neither estimate was statistically significant (TyG: *β* = 30.16, 95% CI −9.32–69.63, *P* = 0.134; METS-IR: *β* = 3.71, 95% CI −0.16–7.59, *P* = 0.060; **Table 3**). These exploratory findings were directionally consistent with, but less precise than, the primary analysis.

**Table 3:** Sensitivity analysis of the associations between novel IR indices and baPWV.

Variables	β (95% CI)	*P*-value
TyG	30.16 (-9.32,69.63)	0.134
METS-IR	3.71 (-0.16,7.59)	0.060

### Subgroup analysis by menopausal status

There was no statistical evidence that menopausal status modified the association of either index with baPWV (P for interaction = 0.222 for TyG and 0.722 for METS-IR). In the fully adjusted analyses, TyG was not significantly associated with baPWV in either premenopausal women (*β* = 18.76, 95% CI −25.68–63.21, *P* = 0.405) or postmenopausal women (*β* = 35.75, 95% CI −23.00–94.50, *P* = 0.231). METS-IR was not statistically significant in premenopausal women (*β* = 4.16, 95% CI −1.22–9.53, *P* = 0.128) and was positively associated with baPWV in postmenopausal women (*β* = 5.68, 95% CI 0.74–10.61, *P* = 0.025). Because the interaction was not significant, the difference in subgroup-specific P values should not be interpreted as effect modification (**Table 4**).

**Table 4:** Associations of the TyG index and METS-IR with baPWV according to menopausal status.

Subgroups	N (%)	Unadjusted model			Fully adjusted model		
		β (95% CI)	*P*-value	*P* for interaction	β (95% CI)	*P*-value	*P* for interaction
TyG							
All patients	306 (100.0)	113.73 (71.12,156.34)	< 0.001		26.66 (-11.49,64.81)	0.170	
Menopausal status				0.704			0.222
Pre-menopause	134 (43.8)	93.82 (48.06,139.58)	< 0.001		18.76 (–25.68,63.21)	0.405	
Post-menopause	172 (56.2)	78.22 (14.85, 141.58)	0.017		35.75 (-23.00,94.50)	0.231	
METS-IR							
All patients	306 (100.0)	8.67 (3.78,13.57)	< 0.001		4.72 (1.03, 8.41)	0.012	
Menopausal status				0.818			0.722
Pre-menopause	134 (43.8)	5.52 (−1.30, 12.34)	0.115		4.16 (-1.22,9.53)	0.128	
Post-menopause	172 (56.2)	6.69 (0.63, 12.75)	0.032		5.68 (0.74,10.61)	0.025	

The unadjusted model represents crude univariable linear regression. For the overall population, the fully adjusted model was adjusted for age, menopausal status, SBP, uric acid, LDL-C, and eGFR. In the menopause-stratified analyses, the fully adjusted model was adjusted for age, SBP, uric acid, LDL-C, and eGFR. TyG, triglyceride-glucose index; METS-IR, metabolic score for insulin resistance; CI, confidence interval.

## Discussion

### Principal findings

In this cross-sectional secondary analysis of 306 women, both TyG and METS-IR were positively correlated with baPWV in unadjusted analyses. After adjustment for age, menopausal status, systolic blood pressure, uric acid, LDL-C, and eGFR, METS-IR remained associated with baPWV, whereas the association for TyG was not statistically significant. The two covariate-adjusted models showed similar exploratory discrimination for elevated baPWV, and menopausal status did not significantly modify either association. In the sensitivity analysis, both estimates were attenuated and imprecise. These findings support an adjusted association between METS-IR and baPWV in this dataset but do not establish superiority, causal effects, or longitudinal predictive utility.

### Comparison of TyG and METS-IR

Arterial stiffness, as assessed by baPWV, is an established marker of vascular aging and is associated with adverse cardiovascular outcomes (4, 5). Although the hyperinsulinemic–euglycemic clamp remains the reference standard for assessing insulin resistance, its invasive nature, technical complexity, and high cost limit its routine clinical use. Consequently, non-insulin-based surrogate indices, including the TyG index and METS-IR, have received increasing attention in epidemiological and clinical research (10, 11, 17). The TyG index primarily reflects glucolipid metabolism through fasting triglyceride and glucose concentrations, whereas METS-IR additionally incorporates BMI and HDL-C and therefore represents a broader composite of metabolic status.

In the present study, both TyG and METS-IR were positively associated with baPWV in the unadjusted and age-adjusted models. After further adjustment for age, menopausal status, systolic blood pressure, uric acid, LDL-C, and eGFR, METS-IR remained positively associated with baPWV, whereas the association for TyG was attenuated and was no longer statistically significant. This pattern suggests that METS-IR may capture metabolic characteristics related to arterial stiffness that are not fully represented by the TyG index. However, the finding that one association was statistically significant while the other was not does not itself demonstrate a statistically significant difference between the two indices. Moreover, because TyG and METS-IR are expressed on different scales, their regression coefficients cannot be directly compared to infer the relative strength of their associations.

The broader composition of METS-IR may offer a plausible explanation for its retained association with baPWV. In addition to fasting glucose and triglycerides, METS-IR incorporates BMI as a measure of general adiposity and HDL-C as a component of the lipid profile. Previous studies have suggested that indices combining glycemic, lipid, and adiposity measures may provide a more comprehensive representation of metabolic dysfunction than indices based only on glucose and triglycerides (20–22). Comparative studies have also reported associations between METS-IR and cardiovascular or cardio-renal-metabolic outcomes (23). Nevertheless, because BMI is mathematically embedded in METS-IR and was not included as a separate covariate in the primary adjusted model, part of the observed association may reflect the adiposity component of the index. Therefore, the present results support a more persistent adjusted association of METS-IR with baPWV in this dataset but do not establish its superiority over TyG.

This cautious interpretation is also supported by the ROC analysis. The covariate-adjusted models containing TyG or METS-IR showed nearly identical discrimination for elevated baPWV, with AUCs of 0.831 and 0.834, respectively, and the difference was not statistically significant according to the DeLong test. Thus, although METS-IR retained an adjusted linear association with continuous baPWV, the present data do not demonstrate a clinically meaningful improvement in discrimination compared with TyG.

### Menopausal transition and vascular health

Cardiometabolic risk profiles vary according to sex and age (24, 25), and the menopausal transition represents an important period of metabolic and vascular change in women. Declining estrogen levels may contribute to impaired endothelial function, altered lipid metabolism, increased vascular inflammation, and reduced nitric oxide bioavailability. Recent evidence has also linked menopausal status with increased arterial stiffness (6). Consistent with these observations, postmenopausal women in the present cohort were older and had higher systolic blood pressure, fasting blood glucose, uric acid, total cholesterol, triglycerides, LDL-C, TyG, METS-IR, AIP, and baPWV, together with lower eGFR, than premenopausal women.

Despite these differences in baseline metabolic and vascular profiles, the formal interaction analyses did not show that menopausal status significantly modified the association of either index with baPWV. In the fully adjusted subgroup analyses, TyG was not significantly associated with baPWV in either premenopausal or postmenopausal women. METS-IR was positively associated with baPWV in postmenopausal women but not in premenopausal women. However, the interaction between METS-IR and menopausal status was not statistically significant (P for interaction = 0.722). Therefore, the difference between the subgroup-specific P values should not be interpreted as evidence that the association was present only after menopause.

The absence of a statistically significant interaction suggests that the available data do not provide clear evidence of effect modification by menopausal status. It does not, however, demonstrate that the associations are identical across reproductive stages. The subgroup estimates had relatively wide confidence intervals, particularly after multivariable adjustment, and the study may have had limited precision for detecting interaction effects. Accordingly, these findings should be regarded as exploratory and require confirmation in larger studies with more detailed information on menopausal duration, hormone concentrations, hormone-replacement therapy, and other reproductive factors.

### Pathophysiological mechanisms

The observed association between METS-IR and baPWV is biologically plausible and may reflect several interconnected pathways linking insulin resistance, adiposity, dyslipidemia, and vascular dysfunction. Insulin resistance is often accompanied by compensatory hyperinsulinemia, neurohormonal activation, oxidative stress, and impaired endothelial signaling. Activation of the renin–angiotensin–aldosterone and sympathetic nervous systems may promote sodium retention, increased vascular tone, vascular smooth muscle cell hypertrophy, and extracellular matrix remodeling, all of which can contribute to arterial stiffening (26, 27).

Insulin resistance and chronic hyperglycemia may also facilitate the formation and accumulation of advanced glycation end-products. These products can form cross-links with long-lived extracellular matrix proteins, including collagen and elastin, thereby reducing arterial elasticity. The interaction of advanced glycation end-products with their receptors may further increase oxidative stress and inflammatory signaling, aggravating endothelial dysfunction and vascular remodeling (28, 29). Although the present study did not directly measure insulin concentrations or advanced glycation end-products, these pathways provide potential biological explanations for the relationship between metabolic dysfunction and increased baPWV.

Adiposity-related inflammation may represent another contributing pathway. High BMI is associated with a substantial metabolic disease burden (30), and adipose tissue can release inflammatory mediators, including TNF-α and IL-6, that promote oxidative stress and impair endothelial function (31). Reduced nitric oxide bioavailability may increase vascular tone, while persistent inflammation may promote structural changes within the arterial wall. Because METS-IR incorporates BMI in addition to fasting glucose, triglycerides, and HDL-C, it may summarize several metabolic characteristics related to vascular stiffness. The reported associations of insulin-resistance-related indices with broader cardio-renal-metabolic outcomes are also consistent with this interpretation (32).

Nevertheless, these mechanisms were not directly investigated in the present analysis. METS-IR should therefore be considered an integrated metabolic marker rather than evidence of a specific causal pathway. Prospective mediation studies incorporating insulin, inflammatory biomarkers, adiposity measurements, endothelial function, and sex hormones are needed to clarify the biological pathways underlying the observed association.

### Clinical implications and sensitivity analysis

METS-IR can be calculated from routinely available fasting glucose, triglyceride, HDL-C, and BMI measurements and therefore has practical advantages for epidemiological research and preliminary metabolic assessment. The retained association between METS-IR and continuous baPWV in the primary adjusted analysis suggests that it may help identify metabolic profiles associated with greater arterial stiffness in women. However, the current results should not be interpreted as demonstrating that METS-IR is a validated screening, diagnostic, or longitudinal risk-prediction tool.

The sensitivity analysis provides an important qualification to the primary finding. After participants with either fasting blood glucose >126 mg/dL or systolic blood pressure >140 mmHg were excluded, the estimated associations remained positive, but neither TyG nor METS-IR was statistically significantly associated with baPWV. The adjusted coefficient for METS-IR was 3.71 cm/s per unit increase, but its 95% confidence interval crossed zero (−0.16 to 7.59; P = 0.060). Thus, the sensitivity analysis was directionally consistent with the primary analysis but did not confirm a statistically robust independent association in the restricted sample. The attenuation may reflect the smaller sample size, reduced exposure variability, exclusion of participants with stronger metabolic or hemodynamic abnormalities, or residual confounding.

Similarly, the ROC analysis showed comparable discrimination between the TyG and METS-IR models, without a statistically significant difference in AUC. These findings indicate that the adjusted association observed for METS-IR does not necessarily translate into superior classification of elevated baPWV. Accordingly, the present results do not justify replacing TyG with METS-IR in routine cardiovascular risk assessment or using METS-IR alone to guide pharmacological treatment.

Lifestyle interventions targeting obesity, insulin resistance, dyslipidemia, and other modifiable cardiovascular risk factors remain important for vascular health (33). However, whether interventions guided by METS-IR can reduce arterial stiffness or improve cardiovascular outcomes cannot be determined from this cross-sectional analysis. Prospective studies with external validation, repeated measurements, and incident cardiovascular outcomes are required before the clinical utility of METS-IR can be established.

## Limitations

This study has several limitations. First, its cross-sectional design precludes causal or temporal inference. Second, the analysis included only 306 women from a single Japanese hospital, which limits generalizability and statistical precision, particularly for the subgroup, spline, sensitivity, and ROC analyses. Third, detailed smoking exposure, physical activity, dietary intake, medication use, and reproductive or hormonal factors were not available, and residual confounding is possible. Fourth, serum insulin and sex hormones were not measured. Prospective, multicenter, and multi-ethnic studies are required to evaluate temporal associations and clinical utility.

## Conclusion

In this cross-sectional dataset of women, METS-IR retained an adjusted association with baPWV, whereas the corresponding association for TyG was not statistically significant. Menopausal status did not significantly modify either association, and the TyG and METS-IR models showed similar exploratory discrimination for elevated baPWV. Prospective external studies are needed to evaluate temporal associations and clinical utility.

## Data Availability

The dataset analyzed in this study is publicly available in the Dryad Digital Repository at https://doi.org/10.5061/dryad.m484p. Further inquiries can be directed to the corresponding authors.
